# Low-Dose CT in the Age of Artificial Intelligence: Techniques, Evidence, and Clinical Impact

**DOI:** 10.3390/tomography12100139

**Published:** 2026-09-22

**Authors:** Abdulaziz A. Qurashi

**Affiliations:** Department of Diagnostic Radiology, College of Applied Medical Sciences, Taibah University, Madinah 42353, Saudi Arabia; aaqurashi@taibahu.edu.sa

**Keywords:** low-dose CT, photon counting CT, deep learning reconstruction, convolutional neural network, iterative reconstruction, hallucination, lung cancer screening, pediatric CT, follow-up imaging

## Abstract

Low-dose computed tomography (LDCT) is essential for reducing potential radiation-associated risks, but increased noise and artifacts can compromise diagnostic confidence. This review examines how AI is reshaping LDCT by improving reconstruction, denoising, protocol optimization, and workflow integration. It outlines the evolution from FBP to IR and then to deep learning methods that better preserve anatomical detail while reducing noise. The review also considers emerging applications in photon-counting CT (PCCT) and discusses clinical use in screening, pediatric imaging, oncology, cardiac imaging, and emergency care. These developments may support safer imaging, improve consistency across studies, and reduce the need for repeat examinations. However, important challenges remain, including limited generalizability across scanners and patient populations, the potential loss of subtle findings, changes in quantitative imaging features, and the need for careful validation before widespread clinical adoption. Further research should focus on multicenter validation, task-based performance assessment, and integration into routine clinical workflows.

## Simple Summary

Artificial intelligence (AI), particularly deep learning-based reconstruction (DLR), can improve image quality and support dose reduction in selected low-dose computed tomography (CT) applications, including lung cancer screening, pediatric imaging, oncologic follow-up, and cardiac CT. Compared with filtered back projection (FBP) and iterative reconstruction (IR), DLR often reduces noise and improves reader confidence while preserving a more natural texture. However, benefits are heterogeneous and depend on the reconstruction algorithm, strength, dose level, scanner platform, and diagnostic task. At ultra-low-dose (ULD) or with strong settings, DLR may suppress subtle findings or generate non-existent structures and can alter radiomic features and downstream AI performance. Evidence for diagnostic accuracy, patient outcomes, and cost-effectiveness remains limited and often single-center or vendor-specific. Broader adoption therefore requires indication-specific, dose-specific, and multicenter validation, task-based performance assessment, and ongoing quality assurance with clear procedures for model versioning and reversion to conventional reconstruction when needed.

## 1. Introduction

Computed tomography has become an essential imaging modality in modern clinical practice, supporting diagnosis, staging, treatment planning, and longitudinal surveillance across a broad spectrum of diseases [1]. However, the continued expansion of CT utilization has raised persistent concerns regarding cumulative exposure to ionizing radiation, particularly in pediatric populations, young adults, and patients requiring repeated examinations [2,3]. In this context, low-dose CT has emerged as a central strategy for minimizing radiation burden while preserving diagnostic utility, consistent with the ALARA principle [4]. The challenge has been to maintain image quality and diagnostic confidence as radiation dose is reduced, especially in anatomically complex regions and for low-contrast clinical tasks.

The evolution of reconstruction strategies has been a major determinant of progress in LDCT. FBP, long regarded as the conventional standard, remains computationally efficient but performs poorly at low photon counts because it does not explicitly account for noise statistics or system imperfections [5]. IR methods represented an important advance by incorporating statistical and physical models of image formation, thereby improving noise suppression and enabling moderate dose reduction [5]. Model-based iterative reconstruction (MBIR) extended these principles further through more comprehensive system modeling, permitting more aggressive dose reduction at the expense of increased computational complexity [6]. Although these methods substantially improved the feasibility of LDCT, they also introduced practical limitations related to workflow, processing time, and image texture that influenced clinical adoption [7].

AI has advanced rapidly in recent years, driven by major gains in computing power and increasingly sophisticated algorithms, and is now being applied to many everyday activities, including healthcare [8]. AI consists of a broad range of computational methods designed to perform tasks such as pattern recognition, prediction, and decision support [8]. Machine learning (ML) is a subset of AI in which algorithms learn patterns from data, whereas deep learning is a subset of ML that uses multilayer neural networks to learn hierarchical features directly from data [9]. Although ML encompasses diverse imaging applications, including computer-aided detection (CAD), automated segmentation and quantification, radiomics, and risk stratification, this review focuses principally on DLR and denoising methods for CT dose optimization and image quality preservation. DLR and denoising methods learn data-driven mappings from low-dose images or data to higher-quality images, often restoring signal and reducing noise while preserving anatomical details [10,11]. Compared with conventional iterative approaches, these methods can provide more natural image texture and faster reconstruction in many implementations [12]. In parallel, advances in PCCT have created new opportunities for dose-efficient imaging by improving detector performance and enabling energy-resolved data acquisition [13]. The convergence of AI and advanced CT hardware can therefore reshape the technical and clinical landscape of low-dose imaging [14].

The clinical relevance of these developments extends across multiple domains, including lung cancer screening, pediatric imaging, oncologic surveillance, coronary CT angiography, emergency neuroimaging, and urolithiasis assessment [15,16,17]. In each of these applications, the principal objective is to reduce radiation exposure without compromising lesion detectability, anatomical delineation, or diagnostic confidence. At the same time, the adoption of AI-enabled LDCT raises important challenges related to generalizability, interpretability, regulatory oversight, medicolegal responsibility, and the demonstration of tangible clinical and economic benefits [18]. Furthermore, effective implementation depends not only on reconstruction performance but also on seamless integration into imaging workflows, picture archiving and communication system, and protocol optimization frameworks [19].

This review aims to synthesize the technical foundations, current evidence, and clinical implications of AI-enabled LDCT. The review first traces the historical evolution of reconstruction strategies, then examines modern AI-based reconstruction and denoising methods, followed by emerging applications in photon-counting CT. It then considers the role of these technologies across major clinical indications, before addressing the principal limitations and implementation challenges. Finally, the review discusses workflow integration and protocol optimization, with the goal of providing a structured and clinically oriented perspective on the current and future role of AI in LDCT.

## 2. Search Strategy

This review was conducted as a narrative review and did not follow a systematic-review or PRISMA framework. Searches were performed in PubMed/MEDLINE, Scopus, and Web of Science between January and August 2026 for studies published between 2016 and 2026 that evaluated LDCT techniques, AI-based reconstruction, DLR or denoising, and relevant technical or clinical outcomes. Search terms included combinations of the following terms: “low-dose CT”, “ultra-low-dose CT”, “AI”, “deep learning reconstruction”, “convolutional neural network”, “iterative reconstruction”, “dose reduction”, “lung cancer screening”, “pediatric CT”, and “follow-up imaging”, with the aim of capturing both technical and clinical literature. Search terms were adapted to the syntax of each database, and reference lists of relevant reviews and primary studies were screened for additional articles.

Priority was given to clinical studies, multicenter investigations, and systematic reviews that evaluated diagnostic performance, radiation dose reduction, and image quality, or clinically relevant workflow outcomes of DLR or denoising compared with FBP or iterative reconstruction in LDCT. Particular emphasis was placed on peer-reviewed studies and reviews reporting quantitative dose metrics, objective image quality indices, or reader performance outcomes. Studies were selected according to their relevance to the review scope, methodological rigor, clinical applicability, and citation impact. Technical phantom, simulation, preclinical, and non-human studies were not used to support claims of clinical diagnostic benefit. However, they were selectively included when necessary to describe reconstruction mechanisms, evaluate technical performance, explain potential failure modes, or summarize emerging PCCT and material decomposition methods for which clinical evidence remains limited. Conference abstracts without a full peer-reviewed publication and non-English articles were excluded. Additional references and landmark trials were identified by backward citation tracking of key articles.

Because this was a narrative review, study selection was guided by the author’s judgment regarding relevance to the review’s scope and clinical focus. Consequently, some selection bias is possible, including preferential inclusion of studies with positive or technically favorable results and under-representation of negative or inconclusive findings. To mitigate this risk, the search strategy intentionally encompassed multiple databases, prioritized peer-reviewed clinical and multicenter studies, and incorporated landmark trials and systematic reviews identified through citation tracking. The review also explicitly highlights limitations in the current evidence base, including heterogeneity in scanners, protocols, reconstruction algorithms, and outcome measures, and avoids overgeneralizing findings across indications.

## 3. Historical Evolution of Reconstruction Strategies in CT

### 3.1. Filtered Back Projection (FBP)

Filtered back projection has historically been the standard analytical reconstruction algorithm in CT because it is fast, reproducible, and computationally efficient [5]. In CT, detectors measure X-ray attenuation along many angular paths through the patient, generating projection data (a sinogram). After calibration and logarithmic transformation, each projection is filtered to compensate for the blurring inherent in simple back-projection; the filtered projections are then back-projected across the image matrix to estimate the X-ray attenuation coefficient of each voxel. CT numbers are subsequently derived from these estimated attenuation coefficients relative to water [20].

FBP is based on simplifying assumptions, including accurate projection measurements, adequately sampled acquisition geometry, and a limited influence of noise and physical effects such as scatter, beam hardening, detector response variation, and photon starvation [21]. Unlike statistical or model-based iterative methods, conventional FBP does not explicitly model the Poisson distribution of X-ray photon counts or assign lower weights to highly noisy measurements [5]. Consequently, fluctuations in low-count projection data are amplified by the reconstruction filter and propagated into the reconstructed image. This leads to increased image noise and may also produce streak artifacts, particularly along highly attenuating paths through the shoulders, pelvis, or in patients who are obese [5].

At standard doses, FBP can provide diagnostically acceptable image quality for many clinical tasks [10]. At low-dose settings, however, the combination of reduced photon flux and the absence of explicit statistical noise modeling can degrade low-contrast detectability, lesion conspicuity, and reader confidence [22]. These limitations are particularly relevant when subtle findings must be identified, such as small pulmonary nodules in screening, low-contrast abdominal lesions, or abnormalities in pediatric examinations. These limitations of FBP provided an important rationale for the development of iterative and MBIR methods, which incorporate measurement statistics and/or system models to improve the noise–resolution trade-off.

### 3.2. Hybrid/Statistical Iterative Reconstruction

Iterative reconstruction differs from FBP because it does not apply a single fixed filtering and back-projection operation to the measured projections. Instead, IR uses repeated processing steps to reduce inconsistencies caused by quantum noise and artifacts while preserving structures judged likely to represent true anatomy [23]. The precise implementation varies among vendors and is often proprietary; therefore, IR methods are commonly categorized as hybrid iterative reconstruction (HIR), statistical iterative reconstruction (SIR), or fully MBIR [24]. HIR and SIR methods generally use simplified noise models and processing in the projection-data domain, image domain, or both, whereas MBIR more comprehensively models the acquisition process and repeatedly compares simulated projections from an estimated image with the measured projections [24].

In projection-data-domain processing, the algorithm estimates the expected noise level for each detector measurement. X-ray detection follows approximately Poisson statistics: measurements containing fewer detected photons have greater relative uncertainty than those with high-count measurements [23]. Low photon counts occur more frequently at reduced tube current, after passage through highly attenuating anatomy, and in larger patients [25]. SIR uses this information to identify projection samples that are less reliable and applies adaptive noise reduction or lower statistical influence to them, while preserving more reliable measurements [24]. This is more accurately described as measurement-dependent weighting rather than “voxel-wise weighting,” because the estimated noise variance originates in the projection data; following back-projection, its effects vary spatially in the reconstructed image [5].

In image-domain processing, an initial reconstruction (often an FBP image) is analyzed repeatedly to distinguish likely noise from anatomical edges and structures [26]. Smoothing is applied preferentially in relatively uniform regions, while edge-preserving constraints limit smoothing across boundaries with substantial intensity differences [26]. The resulting image may then be combined with the original image or passed through additional projection-data and image-domain iterations [26]. By avoiding the complete forward model used in MBIR, hybrid methods achieve shorter reconstruction times and are therefore more readily incorporated into routine clinical workflows [24].

Hybrid SIR can substantially reduce image noise compared with FBP at the same acquisition dose; this noise reduction may be used to improve image quality or to support dose reduction for an appropriately validated clinical task [27]. Reported dose reductions relative to local FBP protocols vary widely, ranging from approximately 23% to 76% across studies and protocols, rather than representing a universal 60% reduction [22,28]. However, the reported dose-reduction values and diagnostic outcomes are indicative and study-specific. They vary by vendor/platform, scanner, protocol, anatomy, patient size, comparator, and performance endpoint; therefore, they should not be generalized across clinical indications.

A key limitation is that strong IR settings can alter the noise-power spectrum and image texture. Excessive smoothing may produce a blotchy, plastic-like, or unnatural appearance and can reduce radiologist acceptance despite lower measured image noise [22]. Thus, reconstruction strength should be selected and clinically validated for the intended diagnostic task rather than optimized solely based on standard-deviation measurements of image noise [25].

### 3.3. Model-Based Iterative Reconstruction (MBIR)

Model-based iterative reconstruction is the most computationally intensive category of conventional iterative CT reconstruction [10]. Like SIR, MBIR accounts for the statistical uncertainty of measured projection data; however, it additionally uses a detailed forward model of how an object is converted into detector measurements. Starting from an initial image estimate, the algorithm forward-projects the estimated voxel attenuation values through a representation of the CT acquisition system to generate synthetic projection data [10]. These simulated data are compared with the measured projection data, and the image estimate is updated iteratively to reduce the discrepancy while controlling image noise and preserving relevant anatomical edges [5].

The forward model may incorporate scanner-specific factors that influence the relationship between object attenuation and detector signal. Depending on the implementation, these include X-ray output and spectrum, focal spot size and shape, detector-cell dimensions and response, fan-beam and cone-beam geometry, helical acquisition geometry, image-voxel dimensions, and measurement noise, including quantum and electronic noise [24]. Modeling these factors more accurately reduces the mismatch between the physical acquisition process and the reconstruction model [24].

Mathematically, MBIR estimates an image by minimizing an objective function with two complementary components: a data-fidelity term and a regularization (prior) term [29]. The data-fidelity term penalizes disagreement between the measured projections and those predicted from the candidate image, with measurements weighted according to their estimated uncertainty [29]. Thus, high-noise, low-count measurements exert less influence than more reliable measurements [30]. The regularization term imposes constraints that favor plausible images [30], for example, by promoting local smoothness in homogeneous tissues while preserving edges between structures with different attenuation. The reconstruction strength controls the balance between data fidelity and regularization. Insufficient regularization leaves excess noise, whereas excessive regularization can oversmooth small structures, alter texture, or reduce the conspicuity of subtle low-contrast lesions [30].

By combining detailed system optics, noise statistics, and edge-preserving regularization, MBIR can improve the noise–resolution trade-off compared with FBP and many HIR implementations [31]. It can reduce image noise and selected artifacts at a given acquisition dose or permit dose reduction while maintaining image quality appropriate for a predefined diagnostic task [32]. However, lower numerical noise does not automatically establish equivalent lesion detectability or diagnostic performance; these outcomes require task-specific clinical validation.

Reported MBIR dose-reduction ranges should therefore be interpreted as indicative rather than universal. In low-dose chest CT, one study reported a 79% reduction in dose–length product relative to its reference-dose examination, while MBIR reduced noise and artifacts compared with adaptive SIR [33]. However, this dose reduction reflects specific protocols, reconstruction settings, patient groups, and image-quality criteria and should not be generalized across CT applications.

## 4. Modern AI-Based Reconstruction and Denoising in LDCT

### 4.1. Deep Learning-Based Reconstruction (DLR)

Artificial intelligence has progressed rapidly in recent years, fueled by substantial improvements in computational capacity and more advanced algorithms, and is now extensively applied across healthcare [10]. Machine learning is a subset of AI in which systems improve their performance by learning from data rather than by following fixed, hand-coded rules [34]. In conventional machine learning workflows, domain experts must first select and construct informative features (a step known as feature engineering) and then feed these engineered features into statistical classifiers such as support vector machines or random forests [34]. Identifying the most effective features a priori is difficult and labor-intensive, especially for complex image-analysis problems [35].

DLR for CT can be formulated as learning a mapping from low-dose or otherwise degraded measurements to a high-quality image estimate [20]. In many implementations, the network approximates a regularized inverse of the CT forward model by minimizing a loss function that balances data fidelity with learned image priors [34]. Unlike conventional IR, which uses handcrafted regularization terms and explicit system models, DLR learns statistical and structural priors directly from training data [5]. This enables flexible adaptation to complex noise patterns, scanner-specific artifacts, and diverse anatomical contexts, but it also introduces dependence on the quality, diversity, and representativeness of the training dataset [36]. These characteristics make DLR particularly attractive for LDCT protocols in which both image quality and workflow efficiency are critical. Table 1 summarizes the reconstruction strategies used for LDCT. 

DLR methods can be further distinguished according to the domain in which the neural network operates. Image-domain denoising approaches take a reconstructed low-dose CT image (typically from FBP or IR) as input and output a denoised image; these methods are conceptually similar to post-processing filters and are relatively straightforward to integrate into existing reconstruction pipelines [21,37]. Raw-data (or sinogram-domain) DLR methods operate on projection data prior to image reconstruction, learning mappings that reduce noise and artifacts in the sinogram before analytical or iterative reconstruction is applied [38]. Hybrid DLR approaches combine both domains, for example, by performing initial sinogram processing followed by additional image-domain refinement, or by embedding learned regularizers within an unrolled IR framework that alternates between data-consistency steps in projection space and learned priors in image space [38]. The choice of domain affects the types of artifacts that can be addressed, the degree of physical consistency enforced, and the complexity of integration with scanner hardware and reconstruction software [21].

#### 4.1.1. Network Architectures for LDCT Reconstructions

Early deep learning approaches to LDCT reconstruction and denoising predominantly used 2D convolutional neural networks (CNNs) applied to individual axial slices or small image patches [21,37]. Many of these models employed residual learning, in which the network predicts the difference between the low-dose and reference-dose images (often interpreted as noise and artifacts) and subtracts this prediction from the input. This formulation stabilizes training and helps preserve low-frequency anatomical information while focusing learning on high-frequency noise and artifact components [39].

Subsequent work extended these designs to 3D CNNs and hybrid 2.5D architectures that process multiple adjacent slices or volumetric patches to exploit through-plane correlations [40,41]. 3D convolutions can better model the spatial continuity of noise and structures along the *z*-axis, which is particularly relevant for helical CT and thin-slice reconstruction [41]. However, 3D networks increase memory and computational requirements, which has motivated hybrid designs that combine 2D and limited 3D operations or use multi-slice inputs with 2D convolutions [41].

Unrolled or “physics-informed” networks have been developed to more explicitly connect deep learning with IR theory [38]. In these models, a fixed number of IR steps are represented as a sequence of trainable blocks. Each block typically alternates between a data-consistency operation that enforces agreement with measured projections (or a sinogram-like representation) and a learned regularization step implemented by a CNN in image space [38]. This architecture can be viewed as approximating an iterative optimization algorithm in which the regularization term is replaced by a data-driven prior. Such networks can achieve strong performance with fewer iterations than classical methods and can be more interpretable than purely black-box image-domain denoisers, although they still require careful design to ensure stability and generalizability [37].

Generative adversarial networks (GANs) have been explored to improve perceptual realism, particularly at ultra-low-dose levels, where conventional pixel-wise losses can produce overly smooth images [42]. In GAN-based LDCT, a generator network produces denoised or reconstructed images, while a discriminator network learns to distinguish them from reference standard-dose images [42]. The adversarial loss encourages the generator to reproduce texture statistics and fine structures that resemble those of standard-dose CT. Conditional GANs, cycle-consistent GANs, and hybrid GAN–CNN architectures have been proposed to further constrain the mapping and reduce the risk of hallucinated structures [43]. Nevertheless, GAN-based methods raise specific concerns about synthetic textures, the potential introduction of non-anatomical features, and the need for rigorous task-based evaluation before clinical use [43].

More recently, attention mechanisms and transformer-based building blocks have been incorporated into reconstruction and denoising networks to better capture long-range contextual information across the image. Transformer-based models can, in principle, model non-local dependencies more effectively than purely local convolutions, which may benefit tasks such as sparse-view reconstruction or very low-dose denoising. At present, most transformer-based CT reconstruction approaches remain in the research domain, with limited clinical validation compared with CNN-based DLR systems [44].

#### 4.1.2. Training Strategies and Data Requirements

Most deep learning-based reconstruction and low-dose CT denoising models are trained in a supervised fashion using paired low-dose and standard-dose CT datasets, which may be obtained from real clinical protocols or simulated by adding realistic noise and system models to higher-dose acquisitions [45]. The choice between real and simulated training pairs entails trade-offs: real pairs capture true scanner behavior and patient motion but can be challenging to acquire at scale, whereas simulated data are easier to generate but may not fully reflect the complexity of clinical noise and artifacts [46,47]. To address the scarcity of perfectly matched pairs, some studies use hybrid strategies that combine real low-dose data with physics-based augmentation or leverage transfer learning from related reconstruction tasks [48,49].

Unsupervised and self-supervised approaches have also been proposed for LDCT [46]. These methods do not require paired high-dose references; instead, they exploit redundancy in the data, data-consistency constraints, or noise models to learn denoising and reconstruction mappings from low-dose data alone [47]. For example, Noise2Noise and related frameworks train networks to map one noisy realization to another, learning to recover the underlying signal by averaging out uncorrelated noise [48]. While these techniques are attractive in settings where standard-dose reference scans are unavailable, they require careful validation to ensure that subtle low-contrast lesions are not inadvertently suppressed [49].

#### 4.1.3. Loss Functions and Perceptual Image Quality

The choice of loss function strongly influences the visual characteristics and diagnostic utility of AI-reconstructed LDCT images [39]. Conventional pixel-wise losses such as root-mean-square error (RMSE) or mean absolute error favor high peak signal-to-noise ratio but tend to penalize deviations from the average appearance, which can lead to overly smooth or “plastic” textures similar to those seen with strong IR [39]. To better correlate with human perception and radiologist preferences, many LDCT studies incorporate structural similarity index measure (SSIM) losses, multi-scale SSIM, or perceptual losses derived from feature representations in pre-trained networks [50].

In GAN-based frameworks, adversarial loss components encourage the generator to produce images whose texture statistics resemble those of standard-dose CT, while auxiliary tasks such as edge preservation or lesion segmentation can be added as secondary loss terms to protect diagnostically relevant structures [43]. Multi-term loss functions that balance fidelity, structural similarity, and adversarial realism are increasingly used to tailor reconstruction networks to specific clinical tasks, such as lung nodule detection or liver lesion characterization [42,43]. The design and weighting of these loss terms are therefore crucial technical parameters that require close collaboration between imaging physicists, data scientists, and radiologists.

**Table 1:** Summary of reconstruction strategies for LDCT.

Technique	Modeling Approach	Noise Behavior	Dose Reduction	Computational Demand	Typical Clinical Role in LDCT
FBP	Analytical inversion of projection data; assumes noise-free linear system	Noise propagated uniformly, pronounced streaks at low-dose; fine but noisy texture	Limited; often requires standard dose to maintain diagnostic quality	Very low; near real-time	Baseline method; historical standard, still used at standard dose and as reference for validation
Hybrid IR	FBP with statistical noise model; partial system modeling in raw and/or image space	Substantial noise reduction; texture may appear smoother or slightly “plastic” at high strength levels	50–60% * dose reduction versus FBP in stone protocol CT, depending on organ and task [51]	Moderate; seconds to a few minutes per series on modern hardware	Widely deployed first-line LDCT solution; backbone of many lung screening and abdominal protocols
MBIR	Comprehensive system and noise modeling with strong priors; full forward physics model	Strongest noise and artifact suppression; very smooth, highly non-FBP-like texture	Up to 60–80% * dose reduction reported in chest/abdomen, but small lesions may be missed at ULD [6]	High; historically tens of minutes per case, now faster but still heavier than HIR	High-end LDCT and ULD research; selected protocols where maximum dose saving is needed and reconstruction time is acceptable
DLR	Data-driven mapping from low-dose to standard-dose appearance; CNN/GAN/unrolled networks incorporating learned priors	Flexible noise suppression with more natural, FBP-like texture; can preserve edges and low-contrast detail better than IR when well trained	Approximately 43–81% dose reductions have been reported in selected thoracic, abdominal, and cardiac CT studies [15,52,53]. Endpoints vary across studies and should not be extrapolated across indications	Low–moderate; typically, closer to FBP than MBIR on vendor-optimized hardware	Emerging standard for LDCT and ULD protocols; used in pediatrics and high-frequency follow-up imaging

* Reported dose-reduction values and diagnostic outcomes are indicative and study-specific. They vary by vendor/platform, scanner, protocol, anatomy, patient size, comparator, and performance endpoint; therefore, they should not be generalized across clinical indications.

## 5. Clinical Applications of AI-Enabled LDCT

The clinical adoption of AI in CT has expanded rapidly, driven by the need to reconcile rising imaging volumes, stricter radiation-dose constraints, and expectations for fast and consistent diagnostic decisions. LDCT is particularly dependent on robust reconstruction and denoising strategies, making it an ideal domain for AI integration across acquisition, reconstruction, interpretation, and workflow optimization. This section provides an overview of the primary clinical applications of AI in CT and how they contribute to optimizing LDCT outcomes. Table 2 presents the characteristics of representative studies evaluating AI-enabled clinical applications in LDCT.

### 5.1. Lung Cancer Screening

Lung cancer screening is one of the most prominent LDCT applications, with large randomized trials having demonstrated mortality reduction using low-dose chest CT for high-risk smokers [54]. Traditionally, these programs relied on IR-enhanced LDCT protocols that balanced dose reduction with sufficient nodule visibility, but concerns persisted regarding cumulative radiation and inter-reader variability [55].

AI-enhanced LDCT frameworks address these concerns on two levels. First, DLR and denoising stabilize image quality at ULD, preserving nodule detectability, especially for small and subsolid lesions [56]. Second, AI-based computer-aided detection and risk stratification tools can assist radiologists in identifying, characterizing, and following pulmonary nodules, potentially reducing missed lesions and improving consistency [56]. Together, these strategies support safer, more efficient screening programs with lower cumulative radiation burdens.

### 5.2. Pediatric and Young Adult Imaging

Pediatric CT represents one of the most critical use-cases for LDCT because children are substantially more radiosensitive than adults and have a longer remaining lifespan during which radiation-induced malignancies may manifest [2]. Many pediatric conditions, including congenital heart disease, chronic lung disease, oncologic disorders, and postoperative follow-up, require repeated CT examinations, making cumulative dose a key consideration in protocol design [2]. Historically, aggressive tube current and voltage reduction combined with FBP often produced images with excessive noise and streak artifacts, which could undermine diagnostic confidence, particularly for subtle parenchymal or vascular findings [25].

AI-based LDCT methods have the potential to overcome many of the limitations imposed by conventional IR techniques by learning data-driven priors that specifically reflect pediatric anatomy, body habitus, and disease patterns [17]. DLR and AI-based denoising networks trained on paired low-dose and standard-dose pediatric datasets can effectively suppress noise while preserving fine structural detail in the lungs, mediastinum, abdominal organs, and musculoskeletal system [45]. In a pediatric CT phantom study, GE TrueFidelity DLR-H enabled approximately 75% and 50% dose reductions for chest and abdominal protocols, respectively, compared with adaptive statistical iterative reconstruction (ASiR)-V-reconstructed reference protocols, while maintaining acceptable reader-rated image quality and model observer detectability for simulated tasks [57]. These findings align closely with the ALARA principle and international initiatives such as Image Gently, which emphasize minimizing radiation exposure in children without compromising diagnostic performance [58]. However, these findings should be interpreted carefully because they relate to technical image quality and reader acceptability rather than disease-specific diagnostic accuracy or patient outcomes.

From a technical perspective, pediatric LDCT places specific demands on AI models. Children exhibit a wide range of body sizes, from neonates to adolescents, and often have higher heart and respiratory rates, leading to greater motion artifacts and variable attenuation patterns. Training datasets must therefore encompass diverse age groups, scanner settings, and clinical indications to ensure that DLR models generalize across the full pediatric spectrum [20]. In addition, the relatively small size of pediatric structures means that subtle denoising errors such as over-smoothing of low-contrast regions or hallucination of nonexistent edges can have a proportionally larger impact on interpretation than in adults [20]. For this reason, task-based assessments focusing on lesion detectability and anatomical conspicuity in pediatric cohorts are particularly important when validating AI-based LDCT protocols.

Operationally, AI-enabled pediatric LDCT can also support protocol standardization and workflow efficiency. AI-driven tools can recommend patient-specific tube current, tube voltage, and scan range based on age, weight, or body surface area, helping technologists consistently achieve acceptable image quality at the lowest feasible dose across different scanners and operators [59]. DLR’s reconstruction speed, which is often closer to FBP than to MBIR, allows for rapid image availability in busy pediatric emergency and oncology settings, where timely decision-making is essential. Furthermore, improved noise suppression can reduce the need for repeat scans due to non-diagnostic image quality, which is particularly beneficial in uncooperative or sedated children [10].

### 5.3. Oncologic Follow-Up and Surveillance

Oncologic patients frequently undergo serial CT for staging, treatment response assessment, and surveillance, often over many years. LDCT is therefore essential to limit cumulative exposure while ensuring consistent quantification of lesion size and density [60]. AI-enhanced LDCT offers improved reproducibility of lesion measurements by reducing noise-related variability, which is particularly relevant for small lesions and subtle changes over time [61]. In addition, AI can aid in automated lesion detection, segmentation, and volumetric analysis, supporting more objective response evaluation and facilitating radiomics research that links imaging phenotypes to tumor biology [62]. Such capabilities may ultimately enable more individualized follow-up strategies that tailor scan frequency and protocol selection to disease dynamics.

### 5.4. Cardiac CT and Coronary Computed Tomography Angiography (CCTA)

Cardiac CT, particularly coronary CT angiography, is another domain where DLR and low-dose protocols intersect [15]. In phantom and clinical CCTA studies, DLR has been shown to improve objective image quality metrics, including lower image noise and higher contrast-to-noise ratio (CNR), and to increase subjective scores for vessel sharpness and overall image quality, with some reports of reduced blooming from calcified plaques and stents [63,64,65]. These improvements are most pronounced at lower tube voltages and in younger or smaller patients, where DLR may enable comparable or improved image quality at reduced radiation dose [66]. In a typical CCTA case, AI-enhanced reconstruction can make borderline segments more interpretable by reducing noise and partial-volume effects, supporting more confident assessment of stenosis severity without increasing exposure [63,66]. However, most evidence to date remains at the level of technical metrics and reader preference; robust task-based lesion detectability, diagnostic accuracy against an appropriate reference standard (e.g., invasive coronary angiography or fractional flow reserve), and patient-level or workflow outcomes have not yet been consistently demonstrated [67].

AI-based post-processing tools further augment CCTA by automating coronary artery segmentation, centerline extraction, plaque characterization, and even computational fractional flow reserve estimation [16]. When combined with LDCT acquisition, these applications may reduce the need for invasive coronary angiography in selected patients, providing comprehensive anatomical and functional assessment from a single low-dose examination.

### 5.5. Neuro and Emergency CT

In emergency neuroimaging, rapid decision making must be balanced against minimizing radiation exposure, particularly in younger patients and those requiring repeated examinations. Deep learning-based reconstruction may improve the technical quality of unenhanced head CT images compared with conventional iterative reconstruction. In a retrospective within-patient study, DLR was associated with lower image noise, higher signal-to-noise ratio (SNR) and CNR, and higher reader-rated image quality scores than adaptive statistical iterative reconstruction (ASIR-V) [68]. Although the DLR examination had a lower volume CT dose index (CTDI_vol_), the overall dose length product (DLP) was slightly higher because the scan range was longer. Importantly, the study evaluated objective and reader-rated image quality rather than task-based lesion detectability or diagnostic accuracy. Therefore, these findings support the potential of DLR to improve image quality under the evaluated acquisition conditions but do not establish reduced total examination dose, preserved sensitivity for urgent pathology, or improved clinical outcomes.

AI triage and detection systems, originally developed for standard-dose CT, can also be applied to LDCT datasets to automatically flag suspected hemorrhage, large-vessel occlusion, or major traumatic findings, prioritizing cases on the radiologist’s worklist [69]. For example, in a busy emergency department, an AI system may automatically highlight a low-dose non-contrast head CT with subtle intraparenchymal hemorrhage, prompting immediate review even before routine reporting queues, thereby supporting faster intervention [70].

### 5.6. Urolithiasis and Other High-Frequency Protocols

Beyond screening and oncology, DLR-LDCT has potential across various applications where repeated or protocolized CT is common. In suspected urolithiasis, for example, ULD non-contrast CT can reliably detect clinically significant stones, and DLR may further reduce radiation dose while preserving stone conspicuity and size estimation [71].

Similar principles apply to other high-frequency protocols, such as LDCT for sinus disease [72], temporal bone evaluation [73,74], or spine assessment in young adults [75]. DLR may help to stabilize image quality across dose levels and body sizes, supporting the routine use of LDCT in protocols that traditionally relied on higher exposure for adequate bone and soft tissue contrast.

**Table 2:** Characteristics of representative studies on AI-enabled LDCT clinical applications.

Author—Year	Study Design	Outcome Parameters	Main Findings	Limitations
(Jiang et al., 2022) [76]	A prospective study of 203 participants who underwent non-contrast ULDCT and standard-dose contrast-enhanced CT (CECT) chest scanning. ULDCT images were reconstructed with FBP, ASIR-V, and DLR (TrueFidelity, GE Healthcare). The conventional-dose CECT image group (ASIR-V level 50%) was used as a reference.	Nodule detection and measurement using DLR nodule assessment system. Three-dimensional segmentation of lung tissue was performed to evaluate image noise.	Compared with standard-dose CECT reconstructed with ASIR-V (level 50%), non-contrast reconstructed with DLR improved the image quality by reducing image noise and increasing the lung nodule detection rate.	Non-contrast CT scans of high and low radiation were not repeated for each participant. Direct comparison between conventional-dose and ULD non-contrast CT was not performed. The DLR nodule detection system was trained based on conventional-dose data sets, not on ULD CT data.
(Wang et al., 2022) [77]	A prospective study included 48 patients who underwent chest examinations with both the standard-dose CT (SDCT) and LDCT. All scans were reconstructed with ASIR-V40%. Additionally, LDCT scans were reconstructed with DLR (TrueFidelity, GE Healthcare) with high-setting (DLR-H) and medium-setting (DLR-M).	Lesion identification, image noise and CNR of thoracic aorta with different reconstruction modes were measured and compared.	DLR significantly reduces image noise in low-dose chest CT scans and DLR-H provides similar image quality as the SDCT ASIR-V40% images with only 4% of the radiation dose.	A lower tube voltage (at 80 kVp) in LDCT than the 120 kVp in SDCT to dramatically reduce radiation dose was used. Small number of patients and only non-contrast chest CT examinations were included.
(Lee et al., 2024) [53]	A prospective study included 296 patients who underwent liver CT scans. All liver CT scans were conducted using a dual-source scanner with the dose split into tubes A (67% dose) and B (33% dose). Blended images from tubes A and B were created using MBIR (ADMIRE, level 3; Siemens Healthineers) to produce SDCT images, whereas LDCT images used data from tube B and were reconstructed with deep learning-based denoising (DLD) (ClariCT.AI; ClariPI).	Image quality and diagnostic capability in detecting malignant liver tumors of LDCT, 33% dose with DLD and SDCT, 100% dose.	Compared with SDCT with MBIR, LDCT with DLD reconstruction allowed for a 67% reduction in liver CT radiation dose while providing significantly lower noise level in the liver and comparable diagnostic performance in detecting malignant liver tumors.	First, only one specific CT scanner from one vendor was used. Second, 10% of malignant liver tumors were clinically diagnosed, which could have affected the readers’ performance.
(Liu et al., 2025) [52]	A retrospective analysis included 100 patients who underwent non-contrast chest CT scans. The experimental group underwent low-dose chest CT at 100 kVp, reconstructed either with high-intensity PI-DL (AI-DLR algorithm developed by Philips Healthcare) (Group A) or conventional iDose IR (Group B) for mediastinal and lung windows. The control group (Group C) received standard-dose CT at 120 kVp reconstructed with iDose, level 4.	Objective image quality metrics, including the mean CT value, standard deviation (SD), SNR, and CNR of the regions of interest (ROIs) in the axial images, were measured and calculated.	Compared to Group C, the volume CT dose index (CTDI_vol_), dose length product (DLP), and effective dose (ED) in the experimental group were reduced by 81%. The subjective image quality scores of Groups A, B, and C were 4.40 ± 0.53, 1.42 ± 0.50, and 4.30 ± 0.51, respectively. The noise level in group A was the lowest, and no significant differences were observed in the SD, CNR, and SNR values between experimental Group A and Group C.	Minor SD differences due to unavoidable ROI positioning/size uncertainty, use of CTDI/DLP with a model-derived k-factor that did not fully account for patient-size-dependent dose, restriction to non-contrast chest CT in healthy checkup patients, and evaluation of only one low-dose protocol (DRI = 4, ~20% of the standard Philips Incisive CT dose).
(Chen et al., 2017) [37]	The study was a method-development investigation that introduced a residual encoder–decoder CNN (RED-CNN) for LDCT denoising. It was trained and evaluated on two datasets: (1) simulated low-dose CT from 165 patients covering multiple body regions, and (2) real clinical full-dose and quarter-dose chest CT images from 10 patients in the Mayo Clinic LDCT Grand Challenge, using patch-based supervised learning and cross-validation across patients.	RMSE, PSNR, and structural similarity index, plus visual assessment of noise suppression, structural detail preservation, and low-contrast lesion detectability on simulated and clinical LDCT images.	RED-CNN outperformed multiple sinogram-, iterative-, and image-domain denoising methods, yielding lower RMSE and higher PSNR/SSIM than other CNNs on simulated data, and providing superior noise reduction, better preservation of vessels and liver lesions, and higher reader scores for low-contrast lesion detectability on clinical quarter-dose chest CT.	The patch-based training with constrained image diversity, the small clinical sample from a single challenge cohort, and reliance on simulated noise that may not fully represent real-world artifacts. Evaluation focused on image quality and lesion detectability, not patient outcomes or multi-vendor generalizability.
(Higaki et al., 2020) [78]	A phantom harboring cylindrical modules with different contrast was scanned on a 320-row detector (Aquilion ONE–family CT scanner). Phantom images were reconstructed with FBP, HIR (AIDR 3D), MBIR (FIRST), and DLR Advanced Intelligent Clear-IQ Engine (AiCE) from Canon Medical Systems.	Image noise, spatial resolution by the 10% MTF, and task-based detectability by a model observer approach.	Compared to other reconstruction methods, DLR produced lower image noise, achieved the highest task-based detectability at reduced dose levels, while offering comparable MTF performance for low-contrast objects.	The dependence on model observers rather than human readers, and evaluation on a single vendor’s CT scanner, limiting generalizability. The study focused on image quality and detectability metrics without assessing clinical outcomes or multi-center performance.
(Benz et al., 2022) [15]	A prospective, within-subject CCTA study in 50 patients who underwent two sequential scans: a SDCT CCTA and a LD CCTA. SDCT datasets were reconstructed with ASIR-Veo (ASiR-V) at 100%, while LD datasets were reconstructed with a DLR algorithm (TrueFidelity, GE Healthcare) at the highest strength level (DLR-H). Quantitative and qualitative comparisons were then made between SDCT/ASiR-V and LDCT/DLR-H images.	Radiation dose (CTDI_vol_, ED), image noise, and quantitative plaque metrics (stenosis severity, composition, volume), plus visual image quality. Agreement between normal- and LD scans was measured using interclass correlation coefficients (ICCs).	Switching from SDCT/ASiR-V 100% to LDCT/DLR-H cut effective dose by about 43% (1.4 to 0.8 mSv) without significantly altering image noise. SDCT and LDCT scans showed excellent agreement (ICC 0.995 for stenosis, 0.974 for plaque composition, with only small plaque-volume differences).	Key limitations included the small, single-center cohort (50 patients), use of only one DLR strength and CCTA protocol on a single vendor’s system, and focus on imaging/plaque metrics without long-term clinical outcomes or evaluation across other vendors and indications.
(Takafuji et al., 2023) [79]	A retrospective, single-center subanalysis of an ongoing prospective cardiac CT protocol, including 58 patients with known or suspected CAD who underwent CCTA on a 320-row scanner (Aquilion ONE Nature Edition) from Canon Medical Systems. Each CCTA dataset was reconstructed twice: with conventional C-DLR (AiCE) and with super-resolution SR-DLR (Precise IQ Engine), and image quality and stenosis grading were compared against invasive coronary angiography (ICA) where available as the reference standard.	Quantitative outcomes included image noise, SNR, vessel and stent edge sharpness. Visual outcomes were segmental stenosis grades (1–5, ≥50% significant), agreement with ICA, and CCTA sensitivity/specificity for significant stenosis with SR-DLR vs. C-DLR.	SR-DLR lowered image noise by up to 31% and increased SNR compared with C-DLR, while improving vessel edge sharpness (21% higher) and yielding thinner stent struts and larger in-stent lumens. It also showed better agreement with ICA stenosis grading (higher weighted κ, including in calcified segments) and generally higher sensitivity/specificity for significant stenosis.	Key limitations were the small, single-center cohort, use of one scanner and vendor-specific DLR algorithms, and evaluation of only a single reconstruction strength for SR-DLR and C-DLR, limiting generalizability and dose-reduction assessment; moreover, SR-DLR was tested only in CCTA with imaging-based stenosis grading, without broader clinical outcome data.
(Pula et al., 2026) [68]	Retrospective, single-center within-patient comparison of 35 patients who underwent unenhanced head CT reconstructed with DLR and ASIR-V. DLR examinations were acquired on a Revolution Apex 256-row scanner, whereas the earlier ASIR-V examinations were acquired on a Lightspeed VCT 64-row scanner closely matched settings but different collimation and gantry tilt capability.	Objective image quality: image noise, SNR, and CNR in predefined brain regions. Reader-rated image quality: overall quality, gray-white matter differentiation, and perceived ability to detect clinically relevant abnormalities. Radiation metrics: CTDI_vol_, DLP and scan length.	Compared with ASIR-V, DLR was associated with higher SNR and CNR and higher reader-rated image quality scores. The mean CTDI_vol_ was 44% lower with DLR than with ASIR-V (52.7 versus 95.4 mGy). However, the mean DLP was 17% higher with DLR (922.8 versus 783.5 mGy·cm), because the DLR examinations had a longer scan range. The findings therefore indicate improved technical and reader-rated image quality at a lower per-slice scanner output, but do not demonstrate reduced total examination dose or preserved diagnostic accuracy.	Retrospective, single-center design, small within-patient cohort, use of different scanner generations and scan geometries, longer DLR scan range due to lack of gantry angulation, and evaluation limited to technical and reader-rated image quality measures. Formal task-based lesion detectability, diagnostic accuracy against a clinical reference standard, and patient-level outcomes were not assessed.
(Otgonbaatar et al., 2025) [80]	A retrospective, single-center diagnostic accuracy study comparing three head CT protocols for intracranial hemorrhage (ICH): SDCT with Advanced modeled IR—HR44 kernel using 192-multidetector row CT (SOMATOM Force, Siemens Healthcare), ULDCT with HIR– FC26 kernel (AIDR-3D, Canon Medical Systems), and ULDCT with DLR (AiCE), both using a 320-multidetector row CT (Aquilion ONE PRISM Edition, Canon Medical Systems). Ninety-three patients who had undergone a standard-dose unenhanced head CT (120 kVp, ~123–188 mA, IR) followed by an ULDCT (120 kVp, 50 mA) reconstructed with both IR and DLR were included, allowing for within-patient comparisons across all three reconstruction strategies.	Quantitative outcomes included radiation dose metrics (CTDI_vol_ and DLP), image noise, SNR, CNR for brain structures. Qualitative outcomes were reader-rated image quality and diagnostic performance for ICH detection, expressed as sensitivity, specificity, and overall accuracy for each protocol.	The ULD CT (120 kVp, 50 mA) achieved about 84% reduction in CTDI_vol_ compared with SDCT (median 5.6 vs. 35.02 mGy). Within this ULD setting, DLR significantly reduced image noise and improved SNR and CNR versus ULD IR and even standard-dose IR. ULDCT with DLR yielded slightly higher sensitivity (99.3% vs. 98.6%) and equal or better specificity (97.5% vs. 97.5%) for detecting intracranial hemorrhage compared with ULD IR, and was statistically non-inferior to SDCT in both image quality and ICH diagnostic performance.	Limitations include the single-center design with a relatively modest sample size, which may restrict generalizability to other institutions and scanner types. The study focused on ICH detection only, without evaluating other intracranial pathologies or broader clinical outcomes such as downstream management or long-term prognosis. It also assessed one ULD setting and specific IR/DLR implementations, so results may not directly extrapolate to other dose levels, vendors, or reconstruction strengths.
(Zhang et al., 2022) [71]	A prospective study evaluated 72 patients with suspected urolithiasis who underwent three unenhanced CT acquisitions in the same session: LDCT reconstructed with HIR (Canon’s HIR technique, AIDR 3D) (reference standard), ULDCT with HIR, and ULDCT with DLR (AiCE, Canon Medical Systems). The study compared stone detection and image quality across the three datasets.	Radiation exposure, urinary-stone detection rate and size estimation, particularly for clinically significant stones ≥3 mm, objective image quality (image noise and SNR) and subjective image-quality scores on a five-point Likert scale.	ULDCT-DLR detected 96.6% of stones, comparable to ULDCT-HIR, while maintaining detection and size assessment of clinically significant stones and improving noise, SNR, and reader-rated image quality. ULDCT reduced effective dose by approximately 77% (1.28 vs. 5.49 mSv; *p* < 0.001)	The study was limited to patients with suspected urinary stones and a single acquisition protocol, so its results may not generalize to other abdominal indications, scanner settings, body sizes, or DLR platforms.
(Otgonbaatar et al., 2025) [72]	A retrospective study included 132 patients who underwent unenhanced ULD paranasal sinus CT for preoperative assessment. Each scan was reconstructed with both DLR (AiCE, Canon Medical Systems) and HIR (Canon’s HIR technique, AIDR 3D), allowing paired image-quality comparison at an effective dose of approximately 0.03 mSv.	Objective measures were image noise, SNR, CNR, noise power spectrum (NPS), and a no-reference perceptual sharpness metric. Radiologists also rated overall image quality, visualization of bony anatomy, and diagnostic confidence for preoperative sinus assessment.	Compared with HIR, DLR substantially reduced noise (28.62 vs. 140.70 HU), increased SNR (22.47 vs. 9.14) and CNR (71.88 vs. 11.81), reduced NPS magnitude/peak, and improved sharpness (0.56 vs. 0.36; all *p* < 0.001). Readers also scored DLR higher for overall image quality, bone visualization, and diagnostic confidence.	Limitations include the retrospective, single-protocol design and comparison of only one DLR and one HIR implementation. The evidence pertains to ULD preoperative PNS CT and image/reader endpoints, so generalizability to other scanners, protocols, sinus pathologies, or diagnostic tasks remains uncertain.
(Boubaker et al., 2024) [73]	An ex vivo cadaveric study evaluated six temporal bones from three adult heads without otologic disease. Ultra-high-resolution CT (UHR-CT) was performed using eight dose protocols that varied tube voltage (80–140 kV) or tube current (20–220 mAs) on a UHR-CT scanner (Aquilion Precision, Canon Medical Systems), with standard HRCT (HRCT; 120 kV, 220 mAs) as the reference using HR-CT scanner (Aquilion One Genesis, Canon Medical Systems). Each acquisition was reconstructed with both HIR (adaptive iterative dose reconstruction, Canon Medical Systems) and DLR (AiCE, Canon Medical Systems).	Radiation dose (CTDI_vol_), objective image noise in the external auditory canal and lateral semicircular canal bone, and reader-based structural visualization. Two blinded head-and-neck radiologists scored seven fine temporal-bone structures on a five-point scale.	DLR improved structural image quality scores by an average of 0.9 points versus HIR and significantly reduced air noise across all UHR-CT protocols. With DLR, UHR-CT at 100 kV/220 mAs or 120 kV/100 mAs maintained effective visualization while reducing CTDI_vol_ by 44% and 58%, respectively, versus conventional HR-CT. At the lowest UHR-CT setting (120 kV/20 mAs), DLR produced image quality comparable to HR-CT with HIR at 4.6 versus 54.2 mGy—a roughly 92% CTDI_vol_ reduction.	The study used only six normal cadaveric temporal bones, limiting clinical generalizability and preventing evaluation of disease detection or diagnostic accuracy. It assessed one manufacturer’s scanners and algorithms, used only helical acquisition, and did not test combined low-kV/low-mAs protocols. Different clinical temporal-bone tasks may also require different image quality thresholds and radiation doses.
(Fujita et al., 2024) [74]	A retrospective single-center study included 36 patients aged 12 years or older who underwent high-resolution temporal-bone CT. Each examination, acquired on one scanner (Aquilion One: Canon Medical Systems), was reconstructed with DLR (AiCE, Canon Medical Systems), HIR ((AIDR) (with FC80): Canon Medical Systems), and FBP FC80 (a kernel for the evaluation of the temporal bone); the analysis directly compared DLR with HIR, with FBP serving as the reference for reader scoring.	Two blinded radiologists rated depiction of temporal-bone microstructures (osseous spiral lamina, cochlear axis, otic capsule, auditory ossicles, and tympanic membrane), noise, and overall image quality on five-point scales. Quantitative endpoints included attenuation of bone and air, image noise, CNR, and edge-rise slope as a sharpness surrogate.	DLR significantly improved subjective depiction of the otic capsule, ossicles, and tympanic membrane, reduced perceived and measured noise, and improved overall image quality compared with HIR. Quantitatively, DLR lowered axial image noise from 39.4 to 32.2 HU and coronal noise from 29.0 to 26.4 HU, increased axial/coronal CNR from 71.8/109.7 to 98.1/136.0, and improved edge-rise slope (2571.5 vs. 2416.0 HU/mm).	The retrospective single-center design, small sample, and inclusion of heterogeneous clinical conditions limit generalizability. DLR performance may not transfer across vendors; inter-reader agreement for air attenuation/noise was limited, and image appearance could have unblinded readers despite formal masking. The study did not test disease-specific diagnostic accuracy, lesion detectability, radiation-dose reduction, or patient outcomes.
(Greffier et al., 2023) [75]	A primarily phantom study of lumbar-spine CT dose optimization, supplemented by illustrative images from three patients with known bone lesions. A 31 cm Mercury 4.0 image quality phantom and an anthropomorphic torso phantom were scanned on a Philips Incisive Premium CT system at four dose settings (DoseRight Index 26, 23, 20, and 15). Images were reconstructed with HIR (iDose4 level 4) and three levels of Precise image DLR algorithm (AI-DLR) (Standard, Smooth, and Smoother). All acquisitions were performed on an Incisive Premium CT system (Philips Healthcare).	Outcomes included CTDI_vol_, NPS, task-based transfer function (TTF), and model-observer detectability for simulated lytic and sclerotic lesions, plus radiologist ratings of noise, smoothing, differentiation, and clinical acceptability.	AI-DLR reduced noise, preserved task-based resolution, improved lesion detectability, and enabled up to 72% dose reduction with clinically acceptable image quality	DLR reduced noise and improved task-based detectability compared with iDose4, especially at the Smooth and Smoother settings, without a material loss of spatial resolution relative to iDose4 at the same dose. Using DRI 15 with the Smooth reconstruction level produced clinically acceptable phantom image quality and lesion detectability while reducing CTDI_vol_ from 19.08 to 5.35 mGy versus the routine DRI-26/iDose4 protocol. Increasing DLR smoothing altered noise texture.

## 6. AI-Enabled Workflow Integration, Protocol Optimization, and Quality Control

The clinical value of AI-enhanced LDCT depends not only on algorithm performance but also on seamless integration into picture archiving and communication system (PACS), radiology information system (RIS), and scanner consoles [19]. In CT, DLR engines are typically embedded within the scanner reconstruction pipeline, enabling reconstructed images to be sent directly to PACS as standard digital imaging and communications in medicine (DICOM) series without additional manual steps [19]. These series are distinguished by specific series description and reconstruction algorithm attributes, allowing PACS hanging protocols to differentiate DLR images from conventional reconstructions.

### 6.1. Advanced AI Applications: Nodule Detection, Organ Segmentation, and Radiomics

More advanced AI applications, such as automated nodule detection, organ segmentation, or radiomic feature extraction, often follow a server-based or cloud-based deployment model [81]. In such architectures, CT images are routed from the scanner or PACS to AI servers via DICOM, processed, and returned as annotated series or structured reports that can be reviewed alongside the original images [81]. Typical pipelines include:

- Automated nodule detection: A common architecture begins with lung segmentation (e.g., using a 3D U-Net or nnU-Net variant) to isolate the lung parenchyma, followed by a 3D convolutional detector that identifies candidate nodules and estimates their size, location, and malignancy probability [82]. Results are post-processed to remove false positives and then encoded as DICOM-structured reports, often using (TID 1500) Measurement Report templates, or as DICOM segmentation objects with nodule masks and bounding boxes [83]. These outputs can be overlaid on CT images in the PACS viewer, with measurements and risk scores available in structured form for downstream reporting and registry integration.
- Organ segmentation: Multi-organ segmentation models (frequently based on nnU-Net or TotalSegmentator-type architectures) are trained on annotated CT volumes to produce voxel-wise labels for structures such as liver, kidneys, lungs, spleen, pancreas, and vertebrae [84]. In practice, DICOM series are converted to a 3D volume for inference, then the resulting label maps are converted back to DICOM and sent to PACS [83]. These segmentations support volumetry (e.g., liver or kidney volume), surgical planning, and radiomics pipelines by providing standardized regions of interest. Recent work has demonstrated that dedicated LDCT-trained nnU-Net models can accurately segment multiple organs on LDCT, enabling automated volumetry and dose tracking in routine practice [85].
- Radiomics feature extraction: Radiomics pipelines typically begin with a segmented volume of interest, followed by standardized pre-processing (resampling to isotropic voxel spacing, intensity discretization, and, where appropriate, Hounsfield unit windowing) [86]. Feature extraction is then performed using tools such as PyRadiomics, which implement image biomarker standardization initiative (IBSI)-compliant pipelines [87]. These pipelines compute first-order statistics, shape descriptors, and texture matrices (e.g., gray-level co-occurrence matrix, gray-level run-length matrix), often after applying filters such as wavelet or Laplacian-of-Gaussian [87]. Extracted features are stored as database records and linked to the corresponding DICOM series via unique identifiers. Because radiomic features are sensitive to reconstruction method, kernel, dose level, and DLR strength, harmonization and reconstruction-specific validation are required before using these features for diagnosis, prognosis, or treatment monitoring [88].

### 6.2. PACS/RIS Workflow Integration and Hanging Protocols

From a workflow perspective, AI should augment rather than disrupt the radiologist’s reading process. Integration strategies that automatically present AI-reconstructed LDCT images and associated annotations within existing PACS worklists and hanging protocols are more likely to be adopted [89]. Concretely, this can be achieved by:

- Configuring PACS to recognize specific series-description or reconstruction algorithm strings for DLR and AI-derived series, and assigning them to dedicated hanging protocols that display DLR images side by side with conventional reconstructions and overlay nodule detections or segmentations [90].
- Using DICOM-structured reporting and DICOM segmentation to convey measurements, nodule lists, and masks in a structured, vendor-neutral format that can be parsed by reporting tools and clinical registries [83].

Despite these advances, interoperability and PACS integration remain practical challenges. AI outputs encoded as DICOM-structured reporting or DICOM segmentation objects are not uniformly supported across all PACS viewers, and some systems still rely on proprietary overlays or secondary capture images, which can limit structured data use and downstream analytics [91,92]. Variability in how vendors implement series description, reconstruction algorithm, and private tags can complicate reliable series identification and hanging-protocol configuration [90]. In addition, firewall, latency, and data-governance constraints often affect server- or cloud-based AI deployments, requiring careful coordination between radiology, IT, and vendors to ensure secure, low-latency integration without disrupting clinical workflow [19].

### 6.3. Protocol Optimization and Quality Control

Machine learning algorithms in CT are not only limited to image reconstruction and detection; they also contribute to workflow optimization and support CT technologists in protocol selection and quality assurance [93]. They can estimate appropriate tube current, tube voltage, and scan range based on patient size, attenuation profiles, and clinical indication, often building on automatic tube current modulation (ATCM) frameworks [94]. Currently available commercial systems primarily implement rule-based or simple model-based ATCM and automatic tube voltage selection methods that adjust exposure according to patient attenuation and predefined noise/image-quality targets; these are now standard on modern CT scanners and are routinely used in clinical practice [95]. Examples include vendor-specific ATCM systems (e.g., SmartmA, CARE Dose 4D, DoseRight, SureExposure) and automatic tube voltage selection tools (e.g., CARE kV, kV Assist) that select tube current and voltage from localizer-based size estimates of patient size and protocol-specific targets [95].

More advanced AI-driven protocol recommendation systems, which use machine learning to suggest or select complete protocols (including scan range, phase selection, and exposure parameters) based on clinical indication, patient habitus, and prior imaging, are beginning to be integrated into scanner consoles and RIS workflow tools but remain less uniformly deployed and, in many cases, are still investigational or early-adoption technologies [8,96]. These systems aim to standardize LDCT techniques across operators and scanners while ensuring that each patient receives the minimum dose consistent with the diagnostic task, but their performance, generalizability, and impact on workflow and outcomes require further multi-center validation [97].

Quality control algorithms can automatically detect technical issues such as motion artifacts, incomplete anatomical coverage, or suboptimal contrast timing, thereby enabling prompt corrective action while the patient remains on the scanning table [98]. For example, in CT pulmonary angiography for suspected pulmonary embolism, a deep-learning algorithm can automatically measure central pulmonary artery enhancement and identify examinations with suboptimal contrast opacification. This automated assessment may help radiology departments detect scanner or protocol-related cause of inadequate enhancement and refine contrast timing protocols to improve diagnostic image quality [99]. Together, these applications illustrate how AI may help integrate low-dose CT principles into routine practice, shifting dose optimization from a one-time protocol-design exercise toward a continuous, case-by-case quality improvement process; however, the extent of clinical benefit remains application-dependent and requires appropriate validation (Figure 1).

## 7. AI and Photon-Counting CT for LDCT

PCCT is an emerging CT technology with potential relevance to low-dose imaging. Unlike conventional energy-integrating detectors, which convert incident X-ray photons into light and subsequently into electrical signals, photon-counting detectors directly register individual X-ray photons and sort them according to their energy [100]. This detector design reduces the contribution of electronic noise and provides energy-resolved data, permitting spectral reconstructions and material-specific image generation from a single acquisition [100]. PCCT has also demonstrated improved spatial resolution and dose efficiency relative to conventional energy-integrating-detector CT in selected applications [101]. For example, early clinical studies and reviews have reported dose reductions of approximately 18–44% in selected abdominal protocols while maintaining comparable technical image-quality endpoints; these estimates are protocol- and task-specific and should not be generalized to all clinical indications [102,103,104,105,106,107].

Deep learning methods for PCCT reconstruction, denoising, and material decomposition are an active area of research [108,109]. PCCT produces multiple energy-bin datasets; however, photon splitting among bins can increase noise in individual energy channels, while detector nonidealities including charge sharing, pulse pileup, and energy cross-talk can distort the measured spectral information [110]. Deep learning methods are being investigated to exploit correlations across energy bins for noise reduction and to improve material-decomposition outputs, including iodine and calcium maps and virtual monoenergetic images [111,112,113]. However, current evidence is predominantly based on phantom, preclinical, and proof-of-concept studies. For example, in preclinical photon-counting micro-CT, a supervised U-net-based denoising method (UnetU) reduced RMSE in matrix-inversion-based material maps relative to both weighted FBP and conventional denoising approaches, achieving the lowest RMSE among the tested methods in iodine (0.200 ± 0.026 mg/mL) and calcium (1.445 ± 0.436 mg/mL) maps; these findings demonstrate technical feasibility rather than validated clinical benefit [114]. Similarly, deep learning denoising approaches have shown faster reconstruction and favorable technical image-quality measures in photon-counting micro-CT experiments, but these preclinical findings should not be interpreted as evidence of improved clinical diagnostic accuracy in human PCCT [114].

Accordingly, PCCT should presently be regarded as a promising platform for dose-efficient, high-resolution, and spectral CT imaging, whereas the additional clinical value of combining PCCT with AI-based reconstruction, denoising, or material decomposition remains to be established. Future studies should compare conventional CT with DLR, PCCT with conventional reconstruction, and PCCT with AI-based processing using matched dose levels and task-specific endpoints. Such studies should evaluate not only technical image-quality metrics but also lesion detectability, diagnostic accuracy against suitable reference standards, quantitative biomarker stability, workflow effects, and performance across vendors, energy-bin configurations, patient populations, and clinical indications.

## 8. Challenges and Limitations in AI-Enabled LDCT

The principal motivation for LDCT is to reduce stochastic radiation risk without compromising diagnostic performance [115]. DLR can substantially reduce image noise and selected artifacts at reduced radiation doses, but it also modifies noise texture, edge sharpness, and low-contrast signal in ways that differ from FBP and conventional IR [116].

### 8.1. Hallucination, Loss of Subtle Lesions, and False Confidence

A first concern is the potential for DLR to generate non-existent structure “hallucinations” or to suppress subtle true findings, particularly at ULD level or when strong reconstruction settings are used [117]. Phantom and reader studies have shown that DLR can preserve or improve low-contrast lesion detectability at moderate dose reductions, but performance remains dependent on the reconstruction algorithm, dose level, and specific diagnostic task [117,118]. At more aggressive dose reductions, small low-contrast lesions may become less conspicuous even when global noise metrics appear favorable [117,119]. In deep learning-based reconstruction, reduced noise or higher SNR/CNR does not necessarily indicate preserved lesion detectability because noise and resolution changes are nonlinear and object dependent. Therefore, DLR assessment should include noise power spectrum and task-based transfer function measurements, together with model-observer or multi-reader task-based studies, rather than relying only on pixel-level noise statistics. Recent work has demonstrated that different DLR implementations vary substantially in their tendency to produce hallucinated structures at low-dose, and that these effects can be algorithm- and dose-dependent [119].

These phenomena are clinically important because DLR images often appear smoother and sharper than FBP or HIR images at comparable radiation doses [120]. This more favorable appearance can increase reader confidence, yet it may coexist with subtle alterations in anatomy or attenuation of faint pathology, creating a classic automation-bias scenario in which readers may over-trust the AI-reconstructed images [121]. In screening and surveillance contexts (e.g., small pulmonary nodules, faint ground-glass opacities, small hypoattenuating liver lesions, or subtle hemorrhagic foci), over-regularized DLR may yield visually acceptable images with reduced conspicuity for the exact abnormalities that drive clinical decisions [117]. Task-based validation, lesion-detection studies, and observer-performance experiments are therefore essential before adopting ULD-DLR protocols in high-stakes applications.

### 8.2. Impact on Downstream AI Tools and Radiomics

A second, closely related limitation is the sensitivity of quantitative imaging and downstream AI tools to reconstruction method, kernel, dose level, and DLR strength [122]. Radiomic texture features are strongly influenced by the underlying noise texture and edge characteristics of CT images [122]. Studies have shown that the reconstruction kernel is often the dominant source of variability in radiomic features, with dose and pitch having comparatively smaller effects [123]. When DLR is introduced, many higher-order texture features change substantially across reconstruction types and strength levels, even when first-order statistics (e.g., mean, median) remain relatively stable [124]. Some DLR implementations have been reported to improve the fraction of stable radiomic features at clinical dose levels compared with FBP or HIR, but stability typically declines at very low doses and across different reconstruction strengths [125].

These findings have direct implications for computer-aided detection, segmentation, and classification models that rely on texture, intensity distribution, or shape features. A model trained on FBP or a specific IR kernel may degrade when applied to DLR images from a different vendor, kernel, or strength setting, or when the scanner model, tube voltage, or software version differs from the intended-use configuration [126,127]. Similarly, radiomics-based biomarkers developed on one reconstruction setting should not be transferred to another without harmonization, image-conversion methods, or separate external validation [126,128]. For example, deep learning-based image-conversion networks have been proposed to reduce kernel-induced variability in radiomic features, but such approaches themselves require validation to ensure they do not introduce new biases or distortions [126,129]. Statistical feature-level harmonization methods, such as ComBat, and image-level or domain-adaptation approaches have improved radiomic-feature reproducibility and selected task-performance measures across reconstruction kernels in phantom and retrospective datasets; however, their clinical efficacy across vendors, acquisition protocols, and prospective real-world settings remains insufficiently established, and they should currently be regarded as investigational rather than standard clinical solutions [130,131].

### 8.3. Generalizability and Domain Shift Across Vendors and Protocols

DLR performance depends critically on the statistical properties of the data used for training, validation, and deployment [18]. Models trained predominantly on a single vendor’s scanners, specific tube voltages, narrow dose ranges, or limited anatomical regions may generalize poorly to other settings [10]. For example, a DLR model trained mainly on adult chest CT at 120 kVp on one vendor’s system may not perform equivalently on pediatric abdominal CT at 80 kVp, on scanners from different manufacturers with distinct detector and reconstruction characteristics, or in the presence of unusual anatomy, motion artifacts, or metallic implants [132,133,134]. Under-representation of rare diseases or certain demographic groups can also lead to biased denoising or reconstruction behavior that disproportionately affects these populations [132,133,134].

This domain-shift problem is exacerbated when DLR is combined with downstream AI tools [135]. A computer-aided detection or radiomics model validated on DLR images from one vendor and dose range may experience reduced sensitivity, increased false-positive rates, or altered feature distributions when deployed on images reconstructed with a different DLR algorithm, kernel, or strength, or on a different scanner model or software version [129,136]. Technical strategies to mitigate these issues include data augmentation, domain adaptation (e.g., adversarial training, feature-space alignment), and inter-vendor harmonization methods, but these approaches remain largely at the research stage for CT reconstruction and require rigorous task-based evaluation before clinical adoption [137,138,139].

From a practical standpoint, institutions should perform local validation using their own phantoms and clinical cases, with emphasis on the intended diagnostic tasks and patient populations. Task-based image-quality assessment, observer studies, and external validation on multi-vendor, multi-protocol datasets are particularly important when training data have limited overlap with local practice. Continuous post-deployment monitoring is also necessary, because changes in scanners, software versions, or protocol settings can gradually erode performance over time. Establishing formal processes for model versioning, retraining, and performance auditing helps ensure that DLR-enabled LDCT remains robust and equitable across the full spectrum of clinical scenarios [140].

### 8.4. Interpretability, Regulatory, and Medicolegal Considerations

Complex DLR models are often perceived as “black boxes,” which can hinder radiologist trust and complicate quality assurance [141]. While interpretability tools such as saliency maps are increasingly used for classification tasks, their application to reconstruction and denoising is less straightforward [142,143]. Radiologists and CT technologists should understand the operational limitations and expected failure modes of DLR (e.g., hallucination risk at ULD, sensitivity to reconstruction strength, and potential impact on quantitative measures) to safely integrate these tools into practice [46,142]. Experience from recent studies suggests that imaging professionals should critically review DLR outputs, especially in high-stakes scenarios or atypical cases, and remain prepared to revert to conventional reconstruction methods when DLR outputs appear inconsistent with raw data or clinical expectations [46].

In many jurisdictions, commercial deep learning-based reconstruction implementations intended for clinical use are subject to applicable medical-device regulatory requirements and must obtain the relevant authorization or clearance from agencies such as the U.S. Food and Drug Administration or corresponding regional regulators before clinical deployment [144]. Regulatory review increasingly emphasizes safety, effectiveness, lifecycle oversight, and post-market surveillance, particularly when performance may change across updates or deployment settings [140]. For DLR-enabled LDCT, regulatory assessment should extend beyond conventional image-quality metrics to include preservation of diagnostic fidelity at reduced dose, lesion-detection performance, and the impact on downstream quantitative applications such as radiomics [145]. This implies that software oversight should address not only direct image interpretation but also secondary quantitative uses, with requirements for reconstruction-specific validation, harmonization strategies, and transparent reporting of technical parameters [144].

Medicolegal responsibility remains complex. In most clinical settings, radiologists are expected to verify DLR outputs and retain ultimate responsibility for diagnostic decisions, while vendors and institutions share obligations for maintaining validated systems, documenting updates, and monitoring real-world performance [140,146]. Ethical concerns regarding privacy, consent for secondary use of CT data in DLR development, and equitable performance across demographic groups further reinforce the need for institutional governance frameworks capable of auditing algorithm behavior, tracking bias, and responding to safety signals after deployment [121]. In practice, institutions implementing DLR-enabled LDCT should establish governance structures that integrate regulatory compliance, local validation, radiomics-aware standardization, and continuous quality assurance to ensure that apparent improvements in image appearance do not come at the expense of diagnostic reliability or quantitative consistency [145].

## 9. Economic Value and Implementation Barriers of AI in Radiology

Artificial intelligence can create economic value in radiology when it improves throughput, reduces repeat or unnecessary imaging, and helps scarce radiology staff work more efficiently [147]. Across published economic evaluations, AI appears most favorable in high-volume or resource-intensive settings, especially when diagnostic performance is at least comparable to that of human readers and the pricing model is fixed rather than usage-based [148]. In health-system ROI modeling, AI platforms have also shown the potential to generate substantial labor-time savings and positive return on investment when they streamline triage, waiting-time management, and reporting workflows [149].

The strongest economic gains tend to come from applications where AI affects the whole care pathway rather than a single reading task. For example, AI can reduce downstream costs by lowering the number of repeat studies, improving follow-up compliance, supporting opportunistic screening, and accelerating access to diagnosis and treatment [150]. AI-based lung cancer screening achieved incremental cost savings of up to 242 USD per patient, while in radiographic screening and radiologist shortage settings, AI was more likely to add value because the saved staff time translated into meaningful operational benefit [148]. These findings suggest that AI is most attractive when it is used to solve bottlenecks rather than replace already fast and inexpensive human workflows.

Another important economic advantage is the potential to improve access and capacity in underserved settings. AI-supported screening programs may be particularly valuable where radiologist shortages are severe, such as tuberculosis screening or other high-volume public health programs [151]. In such contexts, AI can extend specialist reach, reduce the need for double reading, and increase the number of patients processed per day without proportionally increasing staffing costs. Caballo et al. found that an AI-assisted CT acquisition platform reduced examination time by about 40.2% to 52.8% and user interactions by 35.6% to 45.1% [152]. This makes AI economically meaningful not only as a cost-saving tool but also as a capacity-expanding tool.

However, the economic benefit is not always guaranteed, because AI deployment introduces substantial fixed and recurrent costs [153]. These include vendor licensing, graphics processing unit or server infrastructure, PACS integration, cybersecurity, maintenance, upgrades, and local validation, all of which can be expensive and institution-specific [147]. Smaller hospitals may not recover these costs quickly, especially if case volume is modest or if AI is used in only a limited set of CT indications [147].

There are also uncertainty and adoption risks. Savings from fewer repeat examinations or faster reading can be offset if the tool performs inconsistently across scanners, requires frequent revalidation, or creates workflow friction during integration [154]. If the software improves appearance but does not clearly improve meaningful clinical outcomes, then the return on investment may be weaker than expected [155]. For this reason, the most defensible economic argument is not that AI automatically saves money, but that it may do so when deployed in high-volume settings with clear use cases, strong integration, and measurable reductions in repeat imaging, turnaround time, and downstream inefficiency.

The current literature therefore supports a cautious but optimistic interpretation. AI in radiology can produce real economic value, particularly in high-throughput screening, workforce-shortage settings, and integrated workflow platforms, but this value depends on local implementation, pricing strategy, and sustained performance. Future evaluations should move beyond diagnostic accuracy alone and include cost-effectiveness, return on investment, productivity, access to care, and long-term maintenance costs.

## 10. Future Directions and Emerging Trends in AI-Driven LDCT

Federated learning frameworks, which enable collaborative model training across institutions without centralizing raw patient data, offer a promising avenue to improve generalizability while preserving privacy [156]. For CT, such approaches could help create robust AI reconstruction and denoising models that perform consistently across different vendors, protocols, and populations, including rare pathologies and pediatric cases.

Multimodal AI models that integrate LDCT with clinical, laboratory, pathology, and genomic data may support more precise risk stratification, treatment selection, and personalized follow-up strategies [157,158]. In lung cancer screening, for instance, combining imaging features from AI-enhanced LDCT with smoking history, biomarkers, and genetic profiles could refine individual risk estimates and tailor screening intervals [159].

Real-time, AI-guided CT acquisition is another emerging direction [97]. Reinforcement learning and closed-loop control systems, already explored in MRI sequence optimization, could be adapted for CT to automatically adjust scan parameters dynamically in response to measured attenuation, motion, or clinical priorities [141]. Over time, adaptive software as a medical device frameworks will require dynamic regulatory oversight to balance innovation with safety as AI models evolve through continuous learning [160].

Finally, patient-facing AI tools, such as chatbots for pre-scan counselling and education, and decision aids for shared decision-making around screening and follow-up imaging, may complement technical advances in LDCT [161]. Indeed, the successful future of AI in LDCT will depend on multidisciplinary collaboration among radiologists, CT technologists, medical physicists, data scientists, vendors, regulators, and healthcare administrators.

## 11. Conclusions

AI-based methods can improve the technical quality of selected low-dose CT examinations, particularly by reducing image noise and supporting more favorable noise texture, contrast-to-noise ratio, and reader-rated image quality. DLR and denoising have also shown promising task-specific results in selected applications, including lung nodule assessment, pediatric CT, oncologic imaging, cardiac CT, and other protocolized examinations. However, these benefits are heterogeneous and should not be interpreted as evidence that DLR uniformly permits a particular dose reduction or preserves diagnostic performance across all clinical settings. Outcomes depend on the reconstruction algorithm and strength, acquisition dose, tube voltage, patient habitus, anatomical region, diagnostic task, scanner platform, and comparator reconstruction.

At present, the most defensible role for DLR-enabled LDCT is as a validated, indication-specific optimization tool rather than a universal replacement for FBP or IR. Selected applications may be ready for routine use when the DLR implementation, dose range, patient population, and diagnostic task have been locally assessed and shown to provide acceptable image quality and task performance. In contrast, ULD protocols, rare or subtle abnormalities, quantitative imaging and radiomics, PCCT applications, and downstream AI tools require additional evaluation. Technical improvements in noise or image appearance alone are insufficient to establish clinical equivalence, diagnostic safety, or patient benefit.

Broader clinical adoption should therefore be supported by indication-specific, dose-specific, vendor-specific, and multicenter validation, including task-based lesion detectability, diagnostic accuracy against appropriate reference standards, quantitative measurement stability, and assessment across relevant patient populations. Implementation should also include documented software and model versions, predefined quality-assurance measures, monitoring for performance drift and failure modes, and procedures for revalidation or reversion to conventional reconstruction when necessary. Future approaches such as federated learning, multimodal modeling, and AI-guided acquisition may improve generalizability and workflow integration, but these technologies remain investigational for many LDCT applications. Their clinical value should be judged by reproducible diagnostic and patient-level outcomes, not solely by visual quality, numerical noise reduction, or theoretical dose-reducing potential.

The economic case is similarly promising but not yet definitive. AI-enabled LDCT may reduce repeat imaging, improve throughput, and support capacity in high-volume services, but these potential benefits must be weighed against licensing, infrastructure, integration, maintenance, monitoring, and revalidation costs. Formal cost-effectiveness and implementation studies remain necessary to determine when AI-enabled LDCT produces measurable value in particular healthcare systems. Overall, the evidence supports cautious, monitored, and locally validated adoption in selected use cases, not the assumption of uniform readiness across all indications. 

## Figures and Tables

**Figure 1:**
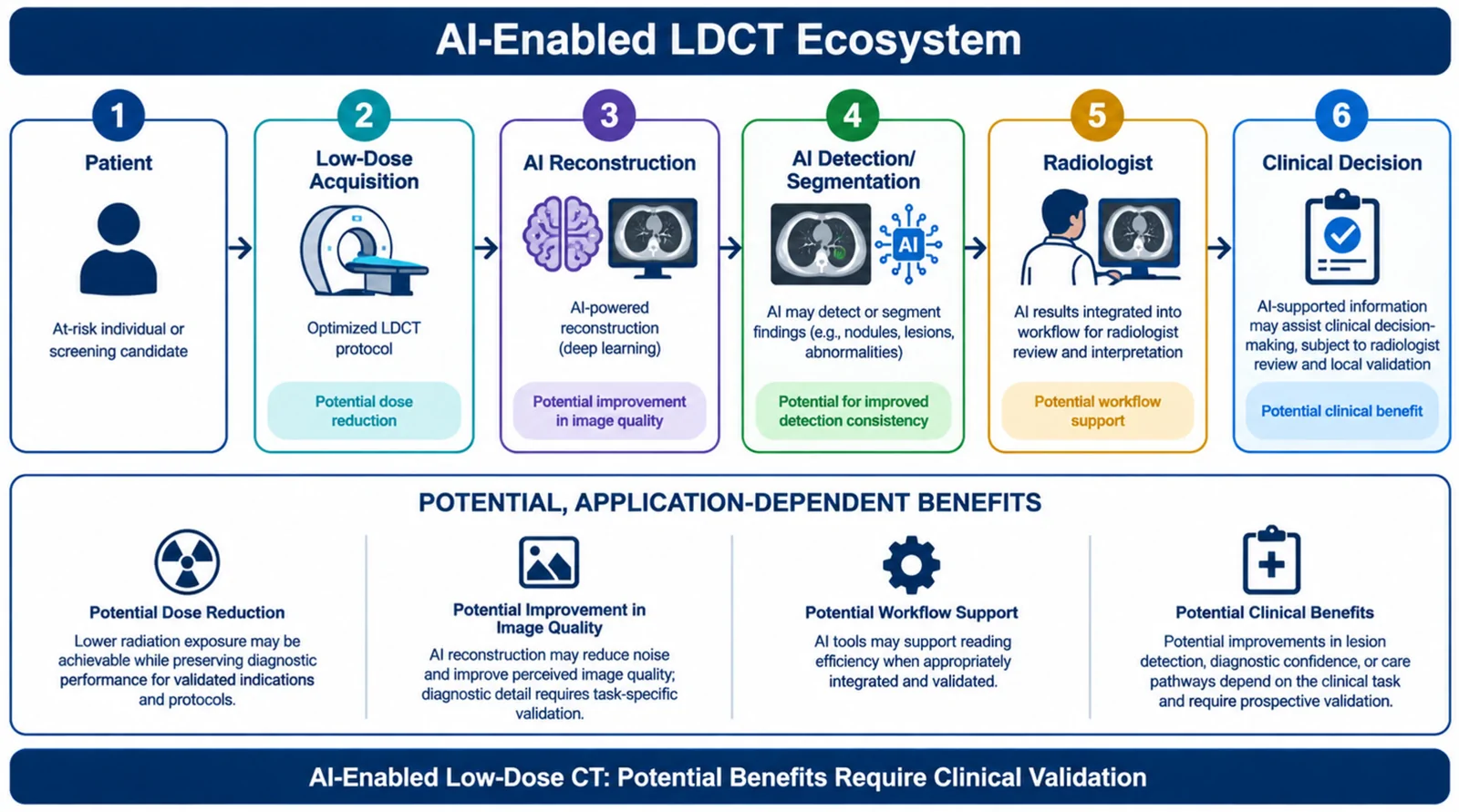
AI-enabled Low-Dose CT Ecosystem: An optimized low-dose CT acquisition may be combined with deep learning-based reconstruction and AI-assisted detection or segmentation, with results subsequently reviewed and interpreted by the radiologist to support clinical decision-making. Potential benefits include optimized radiation exposure, improved perceived image quality, greater detection consistency, and workflow support.

## Data Availability

No new data were created or analyzed in this study. Data sharing is not applicable to this article.

## Abbreviations

The following abbreviations are used in this manuscript:

AI	Artificial intelligence
ALARA	As Low as Reasonably Achievable
ATCM	Automatic tube current modulation
CAD	Computer-aided detection
CCTA	Coronary
CNNs	Convolutional neural networks
CNR	Contrast-to-noise ratio
CT	Computed tomography
CTDI_vol_	Volume CT dose index
DICOM	Digital imaging and communications in medicine
DLD	Deep learning-based denoising
DLP	Dose length product
DLR	Deep learning-based reconstruction
ED	Effective dose
FBP	Filtered back projection
GANs	Generative adversarial networks
HIR	Hybrid iterative reconstruction
ICA	Invasive coronary angiography
ICC	Intraclass correlation coefficient
ICH	Intracranial hemorrhage
IR	Iterative reconstruction
LDCT	Low-dose computed tomography
MBIR	Model-based iterative reconstruction
MTF	Modulation transfer function
NPS	Noise power spectrum
PACS	Picture archiving and communication system
PCCT	Photon-counting computed tomography
PSNR	Peak signal-to-noise ratio
RIS	Radiology information system
RMSE	Root-mean-square error
SIR	Statistical iterative reconstruction
SNR	Signal-to-noise ratio
SSIM	Structural similarity index measure
TTF	Task transfer function
ULD	Ultra-low-dose
